# Music and autobiographical memory: A new experimental protocol

**DOI:** 10.1111/psyg.12947

**Published:** 2023-02-21

**Authors:** Francesca Centomo, Isaline Mottet Wyttenbach, Alice Bencivenni, Alfredo Raglio

**Affiliations:** ^1^ Service Psychiatrique de l'Age Avancée SUPAA/Centre Leenaards de la Mémoire‐CLM Centre Hospitalier Universitaire Vaudois, CHUV Lausanne Switzerland; ^2^ Unité de Neuropsychologie, Département des Neurosciences Cliniques Hôpitaux Universitaires de Genève, HUG Genève Switzerland; ^3^ Music Therapy Research Laboratory Istituti Clinici Scientifici Maugeri I.R.C.C.S. Pavia Italy

Our identity is defined by the continuous narrative process of everyone's existence; this process comes from remembering past experiences and elaborating them through the filter of each one's values and purposes. Autobiographical memory is the core of the self and is organized into semantic (general representation of self) and episodic (specific personal events, in a specific spatial/temporal context, that can be relived mentally) domains. Autobiographical memory is specifically compromised in Alzheimer's disease (AD): In the early stages, people show difficulties in remembering specific and detailed episodes, while at a later stage, semantic knowledge also starts to be damaged. Recent memories are the first ones to be damaged, followed by older ones, based on the Ribot gradient. Together with memory deterioration, the personality becomes static, weak, and less coherent. Persons with dementia often present anxiety and depression, remembering only negative events and generalizing all of their memories. This overgeneralization is evident especially for positive memories and is linked to an impairment in executive control, but also to rumination processes and to a functional avoidance of negative emotional episodes. The autobiographical memory deficit has a big impact on the image of the self, which becomes more negative.

AD still has no effective pharmacological treatment; this is the reason why interest in non‐pharmacological therapies has been increasing in recent years. The purpose of treatments is to maintain as much as possible of people's cognitive functioning and functional autonomies in daily life. Moreover, the second important purpose is to manage psychological and behavioural symptoms and to improve as much as possible the quality of life for people with dementia and their caregivers.

The best care should integrate different interventions, based on each single case and working on many levels.

Reminiscence means the recall and re‐discussion of past events and experiences, such as by using personal supports (photos, music, personal belongings) that work as cues for recall. In the context of dementia, these interventions are frequently used to communicate with people, but also to reinforce people's sense of identity, to promote well‐being, to improve depressive mood, and to stimulate autobiographical memory.^1^ These interventions, structured or free, can be individualized or can involve small groups. Compared to general reminiscence interventions, life review interventions are usually more structured, divided into organized interviews which recall different moments of life; the order is usually chronological or based on specific topics. The purpose is to rebuild personal history, making it meaningful and important once again. Many protocols and manuals have been created, but as yet there is no scientific support to prove the superiority of one above the others. Encouraging results show an improvement in well‐being, mood, and cognitive functioning.^2^

Also, literature has proved that listening to music that accompanied us in significant moments of our lives brings us back in time, stimulating strong emotions and evoking memories—vivid perceptions of those moments.^3^ Moreover, music is highly rewarding, eliciting strong and pleasant emotions, which can help the coding and consolidation of memories and help recover mnestic tracks.^4^

Listening to a specific piece of music, associated with a particular episode of our life, gives us direct access to that memory, together with a strong vividness. This emotional and mnestic power is even more evident in neurodegenerative people with AD.^5^ This paper describes a combined intervention of life review supported by individualized music listening, proposed to a person with memory deficits related to neurodegenerative AD, associated with anxiety‐depression disorder.

The main objective of this intervention was to improve the abilities to access and recall autobiographical memories, to induce a positive change in the reminiscence itself, to reinforce self‐esteem, and to boost mood and satisfaction in life. Moreover, the hypothesis of a benefit in cognitive functions was explored.

A 79‐year‐old woman with 16 years of schooling was treated with an individualized life review program integrated with a music listening approach. She had a history of anxiety‐depressive disorder and benzodiazepine dependence, and was treated for 10 years in a psychogeriatric service. In 2019, a general cognitive worsening, particularly in the areas of memory and executive functions, was found and a diagnosis of mild neurocognitive disorder due to a neurodegenerative (AD) and psychopathological (anxiety‐depressive disorder) origin, was made.

The patient signed an informed consent statement before inclusion in the study.

The program of life review supported by music comprised a daily program of individualized music listening and 10 weekly interview sessions (Table 1). The music listening program was based on five playlists (delivered weekly to the person) created to match five of this person's life stages in chronological order. The interview sessions were conducted at the end of each week, and started with feedback on the music listening experience and exploring the memories related to each life stage. Sessions 1 and 2 focused on the musical anamnesis and personal information; sessions 3–7 specifically referred to each life stage (childhood, adolescence, adult life, aging, latest period). Session 8 was devoted to a global review of the person's life history. For this purpose, a sixth playlist was created collecting all of the songs the person appreciated the most. Finally, in sessions 9 and 10 a personal audiobook (including narrative and musical components) was created with the person. The choice of songs was based on interviews with the person and her husband, and selected positive and activating pieces.

**Table 1:** Scheme of the intervention with a life review supported by music

Assessment	Musical anamnesis and personal data collection	Interview sessions based on feedback concerning the music listening experience and memories related to each life stage	Interview session concerning a global review of the person's life history	Building a personal book including narrative and musical components	Assessment
T0	Sessions 1–2	Sessions 3–7	Session 8	Session 9–10	T1

A neuropsychological assessment (including cognitive, mood, affective, self‐perception/esteem, and quality/satisfaction of life measures) was administered before (T0) and after (T1) treatment. The post‐intervention assessment revealed an improvement on both the cognitive (increase of autobiographical memories, improvement in each sub‐item of the Test Episodique de Mémoire du Passé autobiographique including global and episodic memory) and emotional levels (Hospital Anxiety and Depression Scale: T0 = 7/21, T1 = 5/21; Generalized Anxiety Disorder scale: T0 = 13/21, T1 = 4/21; Positive and Negative Affective Schedules: T0 = 27/50, T1 = 22/50 and T0 = 30/50, T1 = 18/50). Also, self‐perception/self‐esteem (Subjective Aging Perception Scale global scores: T0 = 35/84, T1 = 49/84), satisfaction and quality of life (Quality of Life in AD: T0 = 27/52, T1 = 31/52; The Satisfaction with Life Scale: T0 = 20/35, T1 = 28/35) clearly improved. The improvement of the main outcome of the study (autobiographical memory) reached a moderate effect (percentage of nonoverlapping data = 80% both in global and episodic memory). The detailed assessment and results are summarized in Table 2.

**Table 2:** Main results of assessments for the intervention with a life review supported by music

Autobiographical memory		Pre‐intervention	Post intervention	PND
TEMPau, 0–17 years old	Global (range 0–16)	11	15	80%
TEMPau, 18–30 years old	Global (range 0–16)	**6**	12	
TEMPau, >30 years old	Global (range 0–16)	**7**	**7**	
TEMPau, last 5 years	Global (range 0–16)	5	12	
TEMPau, recent period	Global (range 0–16)	10	12	
TEMPau, 0–17 years old	Episodic (range 0–16)	**4**	12	80%
TEMPau, 18–30 years old	Episodic (range 0–16)	**0**	8	
TEMPau, >30 years old	Episodic (range 0–16)	**0**	**0**	
TEMPau, last 5 years	Episodic (range 0–16)	**0**	8	
TEMPau, recent period	Episodic (range 0–16)	2	6	
**Self‐perception and self esteem**				
SAPS, total (range 0–84)		35	49	
SAPS, physical self‐concept (range 0–21)		9	15	
SAPS, cognitive self‐concept (range 0–21)		9	9	
SAPS, subjective perception of time (range 0–21)		8	12	
SAPS, subjective perception of social relationships (range 0–21)		9	13	
**Mood and affective scales**				
HADS, anxiety (range 0–21; cut‐off 8)		7	5	
HADS, depression (range 0–21; cut‐off 8)		**9**	**8**	
GAD‐7 (range 0–21; cut‐off 7)		**13**	4	
PANAS, positive (range 0–50)		27	22	
PANAS, negative (range 0–50)		30	18	
**Quality and satisfaction of life**				
QoL_AD (range 0–52)		27	31	
SLS‐5 (range 0–35)		20	28	

Abbreviations: GAD‐7, Generalized Anxiety Disorder scales; HADS, Hospital Anxiety and Depression Scale; PANAS, Positive and Negative Affective Schedule; PND, percentage of nonoverlapping data; QoL‐AD, Quality of Life in Alzheimer's Disease; SAPS, Subjective Aging Perception Scale; SLS‐5, The Satisfaction of Life Scale; TEMPau, Test Episodique de Mémoire du Passé autobiographique.

The results of this work show a positive effect on autobiographical memory, in line with previous studies. Psychological symptoms also slightly improved, considering that this research was conducted during the pandemic period. In line with the hypothesis of the study, an improvement of life satisfaction was reported by the person, leading to a global improvement in the quality of life.

This case report represents the first creation of a protocol concerning a music‐based life review intervention, and a first test of its effect on cognitive, affective, self‐perception, and quality of life measures. Considering the degenerative nature of the person's disease, her history of depression, and the difficult pandemic situation happening during this study, these results can be considered encouraging. Future randomized controlled studies are warranted to investigate the efficacy of this protocol in the psychogeriatric field.

## DISCLOSURE

The authors have no potential conflicts of interest to disclose.

## Data Availability

The data that support the findings of this study are available from the corresponding author upon reasonable request.
